# Knowledge and viewpoint of adolescent girls regarding child marriage, its causes and consequences

**DOI:** 10.1186/s12905-021-01497-w

**Published:** 2021-10-06

**Authors:** Somayyeh Naghizadeh, Mojgan Mirghafourvand, Azam Mohammadi, Marzieh Azizi, Saba Taghizadeh-Milani, Hadiseh Ganbari

**Affiliations:** 1 Department of Midwifery and Reproductive Health, Tehran University of Medical Sciences, Tehran, Iran; 2 Department of Midwifery, Tabriz Branch, Islamic Azad University, Tabriz, Iran; 3 Department of Midwifery, Social Determinants of Health Research Centre, Nursing and Midwifery School, Faculty of Nusrsing and Midwifery, Tabriz University of Medical Sciences, South Shariatie, Tabriz, 513897977 Iran; 4 Department Midwifery, Faculty of Medicine, Tabriz Branch, Islamic Azad University, Tabriz, Iran

**Keywords:** Knowledge, Attitude, Child marriage and adolescent girls

## Abstract

**Background:**

Child marriage is a violation of children’s rights and it exposes them to social isolation and psychological damages. These negative effects are not limited to them and expands to the family and society as well. The present research aimed at determining the knowledge and viewpoint of adolescent girls regarding child marriage, its causes and consequences in the city of Tabriz-Iran, in 2020–2021.

**Methods:**

This cross-sectional study was carried out on 300 adolescent girls who had records in the health centers in the city of Tabriz. The data were collected using the sociodemographic characteristics questionnaire, questionnaire of knowledge, and view of adolescent girls regarding child marriage, and its causes and consequences. The multivariate logistic regression model with adjusting the sociodemographic characteristics was used to determine the relationship between the viewpoint of adolescent girls about child marriage and their knowledge in this regard.

**Results:**

The mean (SD) knowledge about child marriage was 6.70 (3.09) (score range: 0–11). The majority of the girls (85.4%) were against marriage before the age of 18 and only 16 girls (5.1%) of them agreed with marriage before the age of 18. Investigating the viewpoint of adolescent girls regarding the causes of child marriage revealed that the important issue leading to reduction of child marriage can be “intellectual, emotional, social, and economic maturity of girls plus their physical puberty (92.4% agree), increase in the girls’ education (79% agree) and increasing girls’ awareness regarding the consequences of early marriage in schools and media (69.6% agree). Illiteracy or lack of education of parents (64% agree), meeting the emotional needs (59.3% agree), family problems and conflicts (59.6% agree), and lack of exhilaration in daily life, especially in the rural regions and suburbs (58.3% agree) were among the reasons of increase of child marriage. The most important consequence of child marriage from the viewpoint of the girls is deprivation from the chance of education. The results of multivariate logistic regression model by adjusting the possible confounding variables, showed that girls who believed the appropriate age for marriage is under 20 were almost 13 times more likely to agree with child marriage than girls who believed the appropriate age for marriage is over 20 years (aOR = 13.82; 95% Cl 2.61–71.16 *p* = 0.002) and girls who opposed their parents’ decision to marry under the age of 18 were less likely to agree with the child marriage than girls who did not oppose their decision (aOR = 0.09; 95% Cl 0.01–0.63 *p* = 0.016).

**Conclusions:**

The results revealed a good level of knowledge and negative attitude to child marriage among the girls. The girls who disagreed with child marriage were more knowledgeable than the girls who agreed with child marriage. Thus, the increase of the level of knowledge of girls and their families regarding the consequences of early marriage and developing the culture for correcting the cultural beliefs and wrong social beliefs to prevent child marriage can decrease this damage to a great extent.

**Supplementary Information:**

The online version contains supplementary material available at 10.1186/s12905-021-01497-w.

## Background

Child marriage is defined as any official, customary, or unofficial marriage (registered or not registered), in which one or both of the spouses or sexual partners are under the age of 18-year-old [1], and conducted before the girl is physically, physiologically, and psychologically ready to carry out the responsibilities of marriage and childbearing [2]. Child marriage is a violation of human rights and children’s rights and exposes them to domestic violence, sexual abuse, rape, and deprives them of access to education [3].

Even though the age of marriage has gradually increased in many countries, however, this increase is specific to higher-income families [4]. Child marriage, especially in less developed countries is considered a global concern, recent statistics show that more than 700 million women married before the age of 18, and more than one-third of them were under the age of 15 when they got married. There is gender inequality in child marriage and it is seen more in girls [5]. Approximately half of the child marriage occurs in Asia (except for China) and the majority of child marriage is carried out in southern Asia [6]. Western and Central Africa has the second prevalence of child marriage with 41% [7]. In accordance with the results of the Population and Housing Census in 2016, approximately 14% of the population of Iran are between the ages of 10–19 years old [3].

According to the latest census of Iran in 2016, the highest number of marriages for women was in the age group of 20–24 years (28.5%), followed by the age group of 15–19 years with 28.1% and 39,645 marriage events (5.6%) for the age group under 15 is registered for both genders. In this census, 98,927 cases of marriage between women aged 15–19 were registered with men aged 20–24, which is the highest number of registered marriages among all age groups and is equivalent to about 14% of all marriages [8]. On the basis of the studies in this regard, 7.7% of the girls in Tehran and more than 40% of girls in Sistan and Baluchestan undergo forced marriage before the age of 18 years old [9, 10].

The consequences of child marriage are more expansive and it does not merely affect the child. Early marriage is accompanied by lifelong effects on social, emotional, and physical health that can continue up to their adulthood and lead to undesired consequences such as losing educational and occupational opportunities and suffering from psychological disorders including depression, anxiety, social isolation, and an increase of domestic violence [11–14]. Besides, child marriage has negative effects on families and society as well. This can result in an increase in poverty and has negative effects on the health and education sectors of a country [14].

Early marriage also known as the child marriage phenomenon is conducted around the world due to a variety of reasons such as poverty and financial incapacity, development of social network and protecting girls against sexual assault and violence[15], gender inequality, cultural and traditional approaches of human communities, illiteracy, and insecurity [16]. Increasing the awareness and knowledge of the society, especially women, and creating societies with gender equality through the empowerment of girls can change people’s attitudes about the issue of child marriage and this can reduce child marriage in these societies [2]. In accordance with the research by Matlabi et al. [17], in Iran, one of the reasons for adolescent girls’ inclination to marriage at this age is the lack of knowledge and awareness of the consequences and damages of the marriage at the young ages. The results of a study in India in 2017 revealed that 59.6% of girl students have little knowledge regarding early marriage and early pregnancy and more than half of student girls (52.5%) had a relatively good attitude and 47.5% of girls had a good attitude to early marriage and pregnancy [2].

In Iran, the most significant reasons for marrying girls at a young age can be considered in three general categories of social, cultural, economic, and legal reasons. The most important economic factor of being a child marriage is poverty. In the field of social and cultural causes, we can mention the issues of tradition, custom, norms, and family and kinship relations in Iran. In most traditional societies such as Iran, women are considered as honor and dignity. Consequently, in these societies, to preserve honor and dignity, they consent to marry girls at an early age and try to protect them through marriage at an early age [18].

Adolescence is the most dynamic period of growth that approximately includes the second decade of a person’s life and involves completely different conditions than the prior period. The destiny and future of a person directly depend on the proper confrontation with changes and developments in this period [19]. Thus, putting an end to child marriage is important for the rights, health, welfare, and capabilities of these girls in the future [20].

Taking into account that child marriage is a violation of human rights and makes them susceptible to social isolation and psychological damages. These negative effects are not limited to the children and expand to the families and society as well. They can cause an increase in poverty and have negative effects on the health and education sectors in a country [11–14, 21]. Therefore, based on the review of the literature, few studies investigated the knowledge and viewpoint of adolescent girls regarding child marriage. Thus, conducting such studies in the cultural platform of Iran in adolescent girls who are affected by this social phenomenon is crucial. The present research aimed at determining the knowledge and viewpoint of adolescent girls about child marriage and its causes and consequences in the city of Tabriz-Iran in 2020–2021.

## Methods

### Type of study and participants

The present research is a analytical-descriptive cross-sectional study and it was carried out from September 2020 through March 2021 on adolescent girls in the city of Tabriz-Iran between the ages of 14–18 years old. The research setting in the present study was the health centers in the city of Tabriz. There is a lot of ethnic, cultural, and religious diversity in different parts of Iran, including the city of Tabriz, the capital of East Azerbaijan province. The metropolis of Tabriz is the third-largest city in Iran after Tehran and Mashhad. This city is known as a religious city and adheres to traditional customs. Despite the nature and traditional cultural and social values ​​of this society, due to the location of this city in the border region and neighboring countries of Turkey and the Republic of Azerbaijan, new attitudes have been created among the youth of Tabriz. The development of global communications, the modernization, and the influence of Turkish-language satellite networks have been effective in creating such an attitude [22]. So, from this point of view, Tabriz society, which is a combination of tradition and modernity, can be a suitable platform for research on girls’ health.

The inclusion criteria of this study were: People who were interested in participating in the study. Consent of one of the parents for their daughter’s participation in the research, Iranian nationality, resident of Tabriz, and girls with the age of 14–18. The exclusion criteria of this research were: Failure to fill in the questionnaire completely and the unwillingness of the girls or their parents to continue the research. It must be noted that child marriage in the present research, includes any official, customary, or unofficial marriage (registered or not registered), in which one or both sexual partners are under the age of 18 [23].

In order to estimate the sample size in the present research, the formula for the estimation of a ratio was employed. In the present research, the value of Z, with the confidence level of 95%, equaled 1.96, and the value of p on the basis of research by Vandana et al. [2], amounted to 52.5, and by considering d (percission) as equal to the 15% around p (attitude regarding early marriage), the sample size was calculated to be n = 155. According to cluster sampling method and considering design effect equal with 1.5 and 20% attrition in the final sample size, 278 people were calculated. In the present study, 300 girls were assessed.

## Sampling

After obtaining the code of ethics from the Ethics Committee of Tabriz University of Medical Sciences (code: IR.TBZMED.REC.1399.482) and providing an introduction letter from the health center of Tabriz, the respective permit for sampling was obtained. The research environment in the present study was the health centers in the city of Tabriz. Sampling was carried out through cluster random sampling method since the city of Tabriz is a metropolis with diverse cultures. Thus, the present research aimed at providing a proper chance for all adolescent girls to participate in this study. For sampling, first, a list of 83 health centers of the city of Tabriz was created and 18 centers were selected randomly among them. Then, the determined sample size was proportionally divided among the selected health centers. Families with girls between the ages of 14–18 who met the inclusion criteria were selected via the SIB system (an Integrated Health System) in the aforementioned centers. These families were selected randomly and the researchers introduced themselves via a phone call and explained the objectives of the research and in case of the children’s and their parent’s consent asked them to participate in the research and be present in the respective health center on a specific date to receive and complete the questionnaire. Or in case of their consent, they were asked to provide an email or a phone number without their name and the link of the questionnaire was sent to them to fill in the house without any stress and confidentially. The SIB system covers more than 90–92% of the population of the city of Tabriz. Before commencement of the study, the participants were provided with explanations regarding the objectives and methods of research, volunteer participation, preserving privacy, and their right to withdraw during any of the stages of data collection. They were ensured that they are not required to write their name or surname and the confidentiality of their information will be maintained. They were asked to hand over a written informed letter of consent.

## Data collection tools

In the present research, the questionnaire created by the researcher team regarding the sociodemographic characteristics, knowledge, and viewpoint of adolescent girls to child marriage and its causes and consequences were used for the collection of the data.

The sociodemographic information questionnaire included questions about age, marital status, number of siblings, parents’ occupation and education, economic status, the marriage of their parents, siblings, or close relative under the age of 18, appropriate age of marriage for girls, the final person to make the decision regarding a girl’s marriage, disagreement with parents if they decide to girls’ marriage under the age of 18 years old.

The questionnaire of the knowledge of adolescent girls regarding child marriage included 11 items and their answers were “correct, incorrect, I don’t know”. The viewpoint of adolescent girls about child marriage was measured through one item “I like to get married before I am 18 years old” based on a 5-point Likert scale (totally agree, agree, no opinion, disagree, totally disagree). This questionnaire is available as Additional file 1: Appendix.

The questionnaire of the viewpoint of adolescent girls regarding the causes of child marriage included 22 questions and was measured on the basis of the 5-point Likert scale (totally agree, agree, no opinion, disagree, totally disagree). The questionnaire of the viewpoint of adolescent girls regarding the consequences of child marriage included 12 questions and was measured on the basis of the 5-point Likert scale (totally agree, agree, no opinion, disagree, totally disagree).

The validity and reliability of these questionnaires were measured before the commencement of sampling. Two qualitative and quantitative methods were used to assess the content validity of questionnaires. In the qualitative method, the content validity of the questionnaire was assessed by 10 specialists in this field. These people included faculty members of midwifery and reproductive health of Tabriz and Tehran Universities of Medical Sciences. After receiving their opinions, the required modifications were made. In the quantitative method, content validity was assessed using content validity ratio (CVR) and content validity index (CVI). The results indicated that the values of CVR and CVI amounted to 0.88 and 0.84, respectively. The reliability of the present questionnaire was obtained using the internal consistency of Cronbach’s alpha test, which amounted to 0.77 for the knowledge questionnaire, and 0.88 for the questionnaire of the viewpoint of adolescent girls regarding the causes of child marriage, and 0.89 for the questionnaire of the viewpoint of adolescent girls regarding the consequences of child marriage.

### Statistical analysis

The data were analyzed using SPSS, 21. The normality of the data was determined by Kurtosis and Skeweness, all of which had a normal distribution. To report the status of knowledge and viewpoint of adolescent girls, and the causes and consequences of child marriage, the descriptive statistics including frequency (percentage), mean, and standard deviation (SD) were used. To study the relationship between the knowledge and viewpoint of adolescent girls regarding child marriage the One-way ANOVA in the bivariate analysis was used.

In multivariate analysis, a multivariate logistic regression model was used with adjustment of sociodemographic characteristics. For this purpose, the variable of viewpoint of adolescent girls regarding child marriage was transformed into a two-state qualitative variable, and the options “I completely agree” and “I agree” were merged and the options “I have no opinion “, “I disagree”, and “I completely disagree” were merged. The chi-square test was used to determine the relationship between sociodemographic characteristics and viewpoint in bivariate analysis. Then the variables that were related to the viewpoint with *p* < 0.2 were included in the multivariate logistic regression model along with the knowledge variable. The results of multivariate logistic regression were reported as a adjusted odds ratio (aOR) with a 95% confidence interval (95% CI). *P* < 0.05 was considered statistically significant.

## Results

The mean (SD) of girls’ age was 16.63 (1.16) years old. More than two-thirds of the participants (63.3%) were students of public schools. Among them merely parents of 10 participants (3.3%) were divorced. The sociodemographic characteristics of the participants and the questions about child marriage are shown in Table 1.

**Table 1:** The socio-demographic characteristics of the participants and their relationship with viewpoint of adolescent girls about child marriage

Characteristic	Number (%)	Relationship with viewpoint	
		Agreement with child marriage Number (%)	*P* value*
*Age (Year)*			
14	13 (4.3)	1 (6.3)	0.592
15	44 (14.7)	3 (18.8)	
16	68 (22.7)	3 (18.8)	
17	92 (30.7)	7 (43.8)	
18	83 (27.7)	2 (12.5)	
*Education*			
Class 9	42 (14)	2 (12.5)	0.309
Class 10	67 (22.3)	4 (25.0)	
Class 11	79 (26.3)	7 (43.8)	
Class 12	112 (37.3)	3 (18.8)	
*Mother’s Education*			
Illiterate/Elementary school	58 (19.3)	7 (43.8)	0.024
Secondary school	42 (14.0)	3 (18.8)	
High school diploma	129 (43.0)	5 (31.3)	
University	71 (23.7)	1 (6.3)	
*Mother’s Job*			
Housewife	231 (77.0)	16 (100)	0.380
Working at home	54 (18.0)	0 (0)	
Working outside the home	15 (5.0)	0 (0)	
*Father’s Education*			
Illiterate/Elementary school	51 (17.0)	5 (31.3)	0.192
Secondary school	73 (24.3)	5 (31.3)	
High school diploma	100 (33.3)	5 (31.3)	
University	76 (25.3)	1 (6.3)	
*Father’s Job*			
Unemployed	7 (2.3)	0 (0)	0.857
Freelance	167 (55.7)	10 (62.5)	
Employee	93 (31.0)	4 (25.0)	
Laborer	33 (11)	2 (12.5)	
*Child of divorce*			
Yes	10 (3.3)	1 (6.3)	0.427
No	290 (96.7)	15 (93.8)	
*Number of sisters*			
0	155 (51.7)	10 (62.5)	0.668
1	118 (39.3)	5 (31.3)	
≥ 2	27 (8.9)	1 (6.3)	
*Number of sisters*			
0	122 (40.7)	3 (18.8)	0.044
1	149 (49.7)	9 (56.3)	
≥ 2	29 (9.7)	4 (25.0)	
*Economic status*			
Poor	22 (7.3)	2 (12.5)	0.346
Moderate	160 (53.3)	11 (68.8)	
Good	118 (39.3)	3 (18.8)	
*Marital status*			
Single	286 (95.4)	13 (81.3)	0.008
Married	14 (4.6)	3 (18.7)	
*The marriage of parents under the age of 18 years old*			
Yes	62 (20.7)	10 (62.5)	0.001
No	238 (79.4)	6 (37.5)	
*The traditional marriage of parents*			
Yes	227 (75.7)	8 (50.0)	0.046
No	73 (24.3)	8 (50.0)	
*The marriage of siblings under the age of 18 years old*			
Yes	17 (5.7)	1 (6.3)	0.914
No	283 (94.3)	15 (93.8)	
*The marriage of close or distant relatives under the age of 18 years old*			
Yes	201 (67.0)	14 (87.5)	0.155
No	99 (33.0)	8 (12.5)	

*Chi-square tests

More than half of the girls (57.7%) considered the proper age of marriage to be from 21 to 25 years old and approximately 44 participants (14.7%) considered it to be 16–20 years old. Approximately two-thirds of girls (63.7%) stated that they take the final decision regarding their marriage and more than three-fourths of girls (77.0%) stated that they would disagree in case their parents decided that they should get married before the age of 18 years old.

The mean (SD) knowledge of the adolescent girls regarding child marriage amounted to 6.70 (3.09) from the acceptable score ranging from 0 to 11. Among them, 137 students (45.6%) had good knowledge, 110 (36.7%) had moderate knowledge, and 53 students (17.7%) had poor knowledge about child marriage. Investigating the viewpoint of adolescent girls about child marriage showed that merely 16 participants (5.1%) agreed with marriage before the age of 18 years old, 28 participants (9.3%) had no opinion in this regard, and the majority of the girls, 256 participants (85.4%), disagreed with marriage before the age of 18 years old. Besides, 19 participants (6.4%) stated that their families want them to get married before the age of 18 years old, 39 participants (13%) had no opinion in this regard, and 242 (80.6%) participants stated that their parents were against their marriage before the age of 18 years old (Table 2).

**Table 2:** Relationship between knowledge and viewpoint of adolescent girls about child marriage

Variable		Number (%)	Mean of knowledge (SD)	The relationship between knowledge and viewpoint (p)
*Knowledge regarding child marriage (Score)*				*P* < 0.001*
Good (Score: 8–11)		185 (61.7)		
Average (Score: 4–7)		103 (34.3)		
Poor (Score: 0–3)		12 (4.0)		
*Viewpoint regarding child marriage*				
Agree		16 (5.1)	7.28 (2.77)	
No opinion		28 (9.3)	3.14 (2.68)	
Disagree		256 (85.4)	3.56 (2.68)	

*One-way ANOVA

Investigating the viewpoint of adolescent girls regarding the causes of child marriage revealed that the important issue leading to reduction of child marriage can be “intellectual, emotional, social, and economic maturity of girls plus their physical puberty (92.4% agree), increase in the girls’ education (79% agree) and increasing girls’ awareness regarding the consequences of early marriage in schools and media (69.6% agree). Illiteracy or lack of education of parents (64% agree), meeting the emotional needs (59.3% agree), family problems and conflicts (59.6% agree), and lack of exhilaration in daily life, especially in the rural regions and suburbs (58.3% agree) were among the reasons of increase of child marriage (Table 3).

**Table 3:** Adolescent girls’ viewpoint on the causes of child marriage

Variable	Totally agree	Agree	No opinion	Disagree	Totally disagree
1. The economic poverty of the family is one of the main causes of early marriage.	15 (5.0)	92 (30.7)	86 (28.7)	36 (12.0)	71 (23.7)
2. The family agrees to early marriage because of the reduction of one of its members.	12 (4.0)	57 (19.0)	86 (28.7)	46 (15.3)	99 (33.0)
3. The family agrees to early marriage to receive Mehr (marriage portion).	5 (1.7)	35 (11.7)	105 (35.0)	53 (17.7)	102 (34.0)
4. Early marriage in girls is carried out to control sexual behavior and premarital pregnancy.	12 (4.0)	49 (16.3)	118 (39.3)	47 (15.7)	74 (24.7)
5. Early marriage in girls is due to family problems and conflicts.	40 (13.3)	139 (46.3)	65 (21.7)	22 (7.3)	34 (11.3)
6. Girls undergo early marriage to meet their emotional needs.	45 (15.0)	133 (44.3)	55 (18.3)	38 (12.7)	29 (9.7)
7. Girls undergo early marriage to meet their sexual needs.	8 (2.7)	54 (18.0)	94 (31.3)	75 (25.0)	69 (23.0)
8. Early marriage in girls is due to a lack of educational and occupational opportunities for girls in accordance with their families.	29 (9.7)	101 (33.7)	87 (29.0)	43 (14.3)	40 (13.3)
9. In Iran, early marriage is carried out due to religious beliefs.	40 (13.3)	91 (30.3)	76 (25.3)	52 (17.3)	41 (13.7)
10. In Iran, early marriage is carried out due to cultural and traditional causes.	52 (17.3)	91 (30.3)	87 (29.0)	44 (14.7)	26 (8.7)
11. Lack of intellectual-psychological maturity is the most important cause of early marriage in girls.	80 (9.6)	112 (37.3)	68 (22.7)	28 (9.3)	12 (4.0)
12. Early marriage is more prevalent in larger families.	36 (12.0)	117 (39.0)	79 (26.3)	48 (16.0)	20 (6.7)
13. Early marriage is conducted to continue the previous family and tribal marriage (for instance marrying the cousin, etc.).	24 (8.0)	90 (30.0)	103 (34.3)	50 (16.7)	33 (11.0)
14. Early marriage is carried out to increase the independence and capability of girls in life.	18 (6.0)	55 (18.3)	105 (35.0)	61 (20.3)	61 (20.3)
15. Illiteracy or lack of education of parents are among other causes of early marriage in girls.	71 (23.7)	121 (40.3)	37 (12.3)	37 (12.3)	34 (11.3)
16. Early marriage is more common in families that the parent got married at a younger age.	40 (13.3)	86 (28.7)	92 (30.7)	55 (18.3)	27 (9.0)
17.Lack of exhilaration in daily life, especially in the rural regions and outskirts can result in the increase of child marriage.	61 (20.3)	114 (38.0)	77 (25.7)	32 (10.7)	16 (5.3)
18. Mothers are of the opinion that girls must get married earlier and when they are in their teen years.	32 (10.7)	48 (16.0)	58 (19.4)	83 (27.7)	79(26.3)
19. Girls cannot make decisions about their marriage, pregnancy health and rights, and their gender on account of cultural problems and patriarchal beliefs.	47 (15.7)	81 (27.0)	86 (28.7)	41 (13.7)	45 (15.0)
20. The intellectual, emotional, social, and economic maturity of girls is necessary in additionto their physical puberty.	230 (76.7)	47 (15.7)	19 (6.3)	2 (0.7)	2 (0.7)
21. With increase in the girls’ education, early marriage decreases.	160 (53.3)	77 (25.7)	44 (14.7)	12 (4.0)	7 (2.3)
22. Raising girls’ awareness of the consequences of early marriage in schools and the media is an effective way to reduce early marriage.	127 (42.3)	82 (27.3)	75 (25.0)	8 (2.7)	8 (2.7)

The important consequences of child marriage in the viewpoint of girls included deprivation of the chance of education of girls (82.7% agree), causing identity crisis and psychological problems due to improper course of childhood and adolescence (81.3% agree), the increase of the probability of families interference in the marital life and decision-making (76.7% agree), an obstacle to have a social status and appropriate job for girls (74.0% agree), and increase of divorce (73.3% agree) (Table 4).

**Table 4:** Adolescent girls’ viewpoint on the consequences of child marriage

Variable	Totally agree	Agree	No opinion	Disagree	Totally disagree
1. Early marriage in girls is considered a form of gender inequality.	95 (31.7)	92 (30.7)	70 (23.3)	30 (10.0)	13 (4.3)
2. Early marriage deprives girls of their chance for education.	155 (51.7)	93 (31.0)	22 (7.3)	16 (5.3)	14 (4.7)
3. Early marriage can lead to an increase in marital conflicts and dissatisfaction with life.	100 (33.3)	103 (34.3)	73 (24.3)	16 (5.3)	8 (2.7)
4. Early marriage can lead to divorce.	132 (44.0)	88 (29.3)	50 (16.7)	21 (7.0)	9 (3.0)
5. Early marriage in girls can result in an increase in social damages such as addiction.	76 (25.3)	74 (24.7)	109 (36.3)	29 (9.7)	12 (4.0)
6. Early marriage can cause the severe attachment of women to their spouses.	65 (21.7)	83 (27.7)	96 (32.0)	40 (13.3)	16 (5.3)
7. In early marriage the probability of interference of families in the marital life and decision-making increases.	122 (40.7)	108 (36.0)	51 (17.0)	14 (4.7)	5 (1.7)
8. In early marriage, a woman cannot protect herself against the violence of her spouse.	103 (34.3)	97 (32.3)	56 (18.7)	31 (10.3)	13 (4.3)
9. Early marriage can lead to the decrease of a woman’s relationship with her surrounding world and result in her social isolation.	89 (29.7)	110 (36.7)	65 (21.7)	28 (9.3)	8 (2.7)
10. Early marriage is an obstacle against achieving a suitable social status and occupation by a girl.	121 (40.3)	101 (33.7)	42 (14.0)	31 (10.3)	5 (1.7)
11. Due to the improper course of childhood and adolescence, child marriage can lead to identity crises and psychological problems in a person.	129 (43.0)	115 (38.3)	36 (12.0)	16 (5.3)	4 (1.3)
12. If I get married early, I will pursue my studies.	158 (52.7)	65 (21.7)	59 (19.7)	6 (2.0)	12 (4.0)

The results of the chi-square test revealed that there was a statistical relationship between girls’ adolescents’ viewpoint on the child marriage and the variables of mother’s education (*p* = 0.024), father’s education (*p* = 0.192), number of brothers (*p* = 0.044), marital status (*p* = 0.008), parental marriage under the age of 18 (*p* = 0.001), traditional parental marriage (*p* = 0.046), the marriage of distant or close relatives under the age of 18 (*p* = 0.155), appropriate age for marriage (*p* = 0.001), being the final decision maker about girls’ marriage (*p* = 0.014), and disagreement with parents if they decide to girls’ marriage under the age of 18 years old (*p* = 0.004) with *p* < 0.2. These variables, with the knowledge variable, entered the multivariate logistic regression model, and the results, by controlling sociodemographic characteristics as possible confounding variables, showed that girls who believed the appropriate age for marriage is under 20 were almost 13 times more likely to agree with child marriage than girls who believed the appropriate age for marriage is over 20 years (aOR = 13.82; 95% Cl 2.61–71.16 *p* = 0.002) and girls who opposed their parents’ decision to marry under the age of 18 were less likely to agree with the child marriage than girls who did not oppose their decision (aOR = 0.09; 95% Cl 0.01–0.63 *p* = 0.016) (Table 5).

**Table 5:** The relationship between knowledge and sociodemographic characteristics with viewpoint of adolescent girls about child marriage based on the multivariate logistic regression model

Variable	Adjusted odds ratio (95% confidence interval)	*P* value
*Knowledge (Reference: Poor)*	–	–
Good	5.05 (0.51–50.31)	0.167
Average	5.71 (0.61–2.95)	0.125
*Mother’s Education (Reference: University)*	–	–
Illiterate/Elementary school	2.10 (24.26–0.08)	0.654
Secondary school	0.57 (17.53–0.02)	0.748
High school/diploma	1.43 (0.09–21.56)	0.795
*Father’s Education (Reference: University)*	–	–
Illiterate/Elementary school	3.36 (0.13–86.69)	0.464
Secondary school	0.86 (0.04–18.02)	0.926
High school/diploma	1.74 (0.11–7.39)	0.693
*Number of brothers (Reference: ≥2)*	–	–
0	0.75 (0.08–6.80)	0.798
1	0.84 (0.15–4.65)	0.843
*Marital status (Reference: Married)*	–	–
Single	0.32 (0.03–2.91)	0.313
*The marriage of parents under the age of 18 years old (Reference: No)*	–	–
Yes	0.00 (1.12–1.03)	0.998
*The traditional marriage of parents (Reference: No)*	–	–
Yes	2.59 (15.70–0.43)	0.299
*The marriage of close or distant relatives under the age of 18 years old (Reference: No)*	–	–
Yes	1.19 (9.25–0.15)	0.869
*Appropriate age of marriage for girls (Reference: ≥20)*	–	–
< 20	13.82 (73.16–2.61)	0.002
Final decision-maker about the girls’ marriage (Reference: Others*)	–	–
My self	0.55 (0.11–2.89 )	0.483
*Disagreement with parents if they decide to girls’ marriage under the age of 18 years old (Reference: No)*	–	–
Yes	0.09 (0.63–0.01)	0.016

*Father, mother and relatives

## Discussion

The results showed that 45.6% of girls had good knowledge about child marriage.majority of the girls (85.4%) disagreed with marriage before the age of 18 years old. The girls stated that the main reasons for the decrease of child marriage include the intellectual, emotional, social, and economic maturity of girls plus their physical puberty. They also stated that illiteracy or lack of education of parents are the main causes of the increase of child marriage. The most important consequence of child marriage from the viewpoint of the girls is deprivation from the chance of education.

The results revealed that the mean knowledge of the adolescent student girls regarding child marriage amounted to 6.70 (ranging from 0 to 11). About half of them (45.6%) had good knowledge about child marriage. Babiker et al. conducted a study in Al Jazeera. They found that the majority of the participants (77.4%) had perfect knowledge about the negative consequences of child marriage [24]. Vandana et al., in their research in India, manifested that knowledge of school girls regarding early marriage was 14.3 (ranging from 10 to 21) [2]. It did not match the results of the present research. These differences might be due to regional and cultural differences.

The results of the present research demonstrated that the majority of girls (85.4%) disagreed with marriage before the age of 18 years old. The results of a study in India revealed that more than half of school girls (52.5%) had a good attitude and 47.5% had a relatively good attitude regarding early marriage [2]. Sumalatha et al. conducted a study in which 73.3% of participants disagreed with early marriage [25]. The results of the present research did not match the results of this study, which can be due to regional and cultural differences.

More than half of the girls (57.7%), considered the proper age of marriage to be 21–25 years old and approximately 44 participants (14.7%) considered it to be 16–20 years old. In accordance with the latest survey regarding the values and attitudes of Iranians, 47% of the participants stated that the proper age of marriage for girls is between 16 and 20 years old and 39% stated that age to be 21–25 years old [26]. The reason for such differences can be due to the fact that all participants were from the urban regions and this city is considered to be a metropolis in comparison to other cities of this country. However, the results of the survey regarding the values and attitudes of Iranians included all provinces of the country, together with rural regions, nomadic tribes, and outskirts. Thus, the factor of development and the living region has a crucial role in the phenomenon of child marriage.

Investigating the viewpoint of adolescent girls regarding the causes of child marriage revealed that the important issue leading to reduction of child marriage can be intellectual, emotional, social, and economic maturity of girls plus their physical puberty, increase in the girls’ level of education and increasing girls’ awareness regarding the consequences of early marriage in schools and media. Illiteracy or lack of education of parents, to meet the emotional needs, family problems and conflicts, and lack of exhilaration in daily life, especially in the rural regions and suburbs were among the reasons for the increase of child marriage. In accordance with the research by Matlabi et al., in Iran, one of the reasons for adolescent girls’ inclination to marriage at this age is the lack of knowledge and awareness of the consequences and damages of the marriage in the young ages [17]. In a study by Babiker et al., the majority of girls (67.4%) stated that the main reason for child marriage was the traditions [24]. Ahmed et al. carried out a study in Egypt. In their study, they argued that the main causes for early marriage were to prevent promiscuity prior to marriage (35%) and difficulty after marriage (28%). The reasons for the lack of support of child marriage were hurting mothers (26%), difficulty in caring for children (18%), and deprivation of education (18%) [27].

The main consequences of child marriage from the viewpoint of girls in the present research included girls’ deprivation of the chance of education, causing identity crisis and psychological problems due to improper course of childhood and adolescence, the increase of the probability of families interference in the marital life and decision-making, an obstacle to have a social status and appropriate job for girls, and increase of divorce. In accordance with the report by the Islamic Parliament Research Center of The Islamic Republic of Iran, deprivation of education, death caused by pregnancy, lack of sexual consent, domestic violence, lack of observation of children’s rights, forced marriage, lack of intellectual and social maturity are not seen in child marriage more than other types of marriage and it stated that there has been exaggeration in this regard and it is not a critical issue. Thus, in accordance with this report, the legal age of marriage for girls in Iran is considered to be 15 years old [28]. In accordance with the rules and regulations of the international communities including UNICEF, child marriage includes any official, customary, or unofficial mirage, in which one or both of the spouses or sexual partners are under the age of 18-year-old [1]. Thus, the legal age of marriage in Iran is different from other societies and the consequences caused by child marriage in Iran are reported less than its actual measures.

Finally, it is recommended to provide proper education in the different levels of society to increase the knowledge of girls, parents, and social and educational programs to empower girls in order to prevent child marriage.

One of the limitations of the present research is being cross-sectional, due to which the relationship of knowledge and viewpoint in this research cannot be demonstrated as cause and effect relationship. The responses could be biased due to the nature of the questions of the research and the researcher tried to eliminate this limitation by ensuring the participant of confidentiality of their information and completing the questionnaires without mentioning their name and surname. Besides, the present research was conducted in the city of Tabriz, with Azari ethnicity, therefore, its results cannot be generalized to other cities and ethnicities. Also, the lack of coverage of 8–10% of the population of Tabriz by the SIB system who have not referred to health centers in the city and have not registered is considered as a limitation of this study.

## Conclusions

The results of the present research indicated a good level of knowledge and negative attitude to child marriage among the girls. It demonstrated that the girls who disagree with child marriage were significantly more knowledgeable than the girls who agreed with child marriage.

Thus, by increasing the level of knowledge of girls and their families regarding the consequences of early marriage and developing the culture for correcting the wrong cultural and social beliefs to prevent child marriage, this damage can be decreased to a great extent.

## Supplementary Information

**Additional file 1.** Questionnaire of the knowledge of adolescent girls about child marriage.

## Data Availability

Datasets used and analyzed during this study are available from the corresponding author on reasonable request.

## Abbreviations

UNICEF: United Nations International Children’s Emergency Fund
95% CI: 95% confidence interval
SD: standard deviation
GLM: general linear model
