# Supplementary material for: Knowledge and viewpoint of adolescent girls regarding child marriage, its causes and consequences
Source: BMC Womens Health. 2021 Oct 6;21:351. doi: 10.1186/s12905-021-01497-w (PMC8495953; doi:10.1186/s12905-021-01497-w)
Supplement: Supplementary file 1 — Additional file 1. Questionnaire of the knowledge of adolescent girls about child marriage. [file 12905_2021_1497_MOESM1_ESM.docx]

Additional file 1: Appendix

**Questionnaire to assess the knowledge of adolescent girls about child marriage (before the age of 18)**

1. The legal age of marriage for girls in our country is 15 years and older.

True  False  I do not know

1. Early marriage of girls is a human rights violation in the eyes of the international community, including the United Nations Children's Fund.

True  False  I do not know

1. Girls who marry early are more likely to be exposed to domestic violence, physical and sexual abuse.

True  False  I do not know

1. Girls who marry before the age of 18 have not reached mental, emotional, and sexual maturity.

True  False  I do not know

1. Girls who marry before the age of 18 have not developed individual and social skills. True  False  I do not know
2. Girls who get married early are more prone to depression and anxiety.

True  False  I do not know

1. Girls who get married before the age of 18 need their father's permission to achieve their legal rights.

True  False  I do not know

1. Girls who get married early do not have enough knowledge about proper sex.

True  False  I do not know

1. Girls who marry early are more prone to a variety of sexually transmitted diseases, including AIDS.

True  False  I do not know

1. Girls who marry early are more likely to have genital and anal injuries.

True  False  I do not know

1. Girls who marry early are unable to use proper contraception.

True  False  I do not know
